# Labeled photovoltaic installations for orthographic aerial imagery in Queens, New York

**DOI:** 10.1038/s41597-025-06523-2

**Published:** 2026-01-06

**Authors:** Tyler Furedi, Edwin Kimsal, Samara Cornejo, Nicholas Liero, Joseph Ranalli

**Affiliations:** Penn State Hazleton, Hazleton, PA 18202 USA

**Keywords:** Photovoltaics, Energy grids and networks

## Abstract

Obtaining data about rooftop photovoltaic installations presents a challenge for energy researchers. Some research efforts have attempted to utilize computer vision approaches to identify photovoltaic installations from aerial imagery. This dataset consists of manually labeled locations of photovoltaic installations for publicly available aerial imagery of Queens, New York, USA in 2018. The labels comprise 14,000 polygons corresponding to roughly 5,500 separate installations. The median polygon size is 13 *m*^2^, with a total area of close to 380,000 *m*^2^. Researchers may be interested in applying this dataset for the training of deep learning models for computer vision or to investigate deployment of photovoltaics in urban areas. While other similar datasets exist, there are several unique aspects of this location that make it attractive for further study: it encompasses a densely populated, urban environment; imagery contains four channels (three colors, plus infrared); and the source dataset is re-acquired periodically by the state of New York, offering the opportunity for these labels to form the basis of a time resolved study of photovoltaic deployment.

## Background & Summary

Data about rooftop photovoltaic (PV) installations is important for energy researchers, electric utility operators, public policy makers and a variety of other stakeholders with an interest in distributed energy development. Reliable sources of data about these installations may be difficult to obtain^1^, and data availability, quality and format varies significantly across jurisdictional boundaries. Accurate representations of these data remain impactful though, with up to 40% of global PV capacity estimated to be made up of rooftop-scale systems^2^.

Identifying photovoltaic installations from aerial imagery data has been a promising area of research in recent years. Several investigators have demonstrated the ability of neural network-based computer vision approaches to develop PV datasets in literature^3–9^ and published software packages, e.g. Satlas (https://satlas.allen.ai/). A problem of scale exists, where data with large coverage area (e.g. satellite imagery) usually lack sufficient resolution to completely detail rooftop scale systems^10^. Thus, identifying PV on the rooftop scale typically requires the use of aerial data, with pixel resolution sufficiently fine (< 0.3 m/pixel^10^) to fully resolve rooftop scale systems.

Efforts have been made to develop foundation models that could serve as the basis for other deep learning approaches to object identification in aerial imagery^11^. However, investigators have observed that generalizability of PV identification across datasets is difficult, meaning that it is typically necessary to label some local data to train models that reach their maximum performance on a given data set^12–14^. Deep Active Learning strategies have been proposed to reduce the labeling effort while maintaining high levels of performance^15^. In addition to simply segmenting PV images, investigators have also utilized overhead data to extract system metadata (e.g. capacity and orientation) from imagery^16–18^.

As mentioned, the generalizability of trained models for PV identification has proved difficult, and efforts to improve or validate generalizability will necessarily require access to diverse reference data. Global level segmentation datasets for PV exist including those created by Kruitwagen *et al*.^3^ and Li *et al*.^7^ as well as the original DeepSolar^4^ and the updated DeepSolar-3M^9^ (https://github.com/rajanieprabha/DeepSolar-3M). Public datasets also exist with a higher resolution for supporting segmentation of PV at a rooftop scale, including those covering multiple cities in California (USA)^19^, two datasets (from different sources) covering areas of France^20^, two areas of Italy^21^, areas of the Netherlands^22^, China^23^, Southern Germany^24^ and Denmark^25^. The dataset presented in this paper adds to these existing datasets with labels for 2018 aerial imagery of Queens, New York, in the United States. Partial versions of this dataset have been used for training in previous studies^14,15,26,27^. Compared to existing datasets, the data from Queens has a more urban character, featuring comparably fewer open green spaces and many large flat-roof systems that may be useful for studies of generalizability^14^. A summary of information comparing the high-resolution, human-labeled datasets is presented in Table 1.

**Table 1:** Dataset Summary.

Cite	Loc	Res. (m/pix)	Img Type	Annotations
^19^	California	0.3	RGB	~19000
^20^	France	0.1^*^	RGB	~13000
^21^	Italy	0.3	RGBIR	9462
^22^	Netherlands	0.075	RGB	4389
^23^	China	0.1^*^	RGB	3713
^24^	Germany	0.15^*^	RGB	2542
Ours	New York City	0.15	RGBIR	14064

^*^multiple resolutions available, aggregate annotation numbers reported

## Methods

**Fig. 1:**
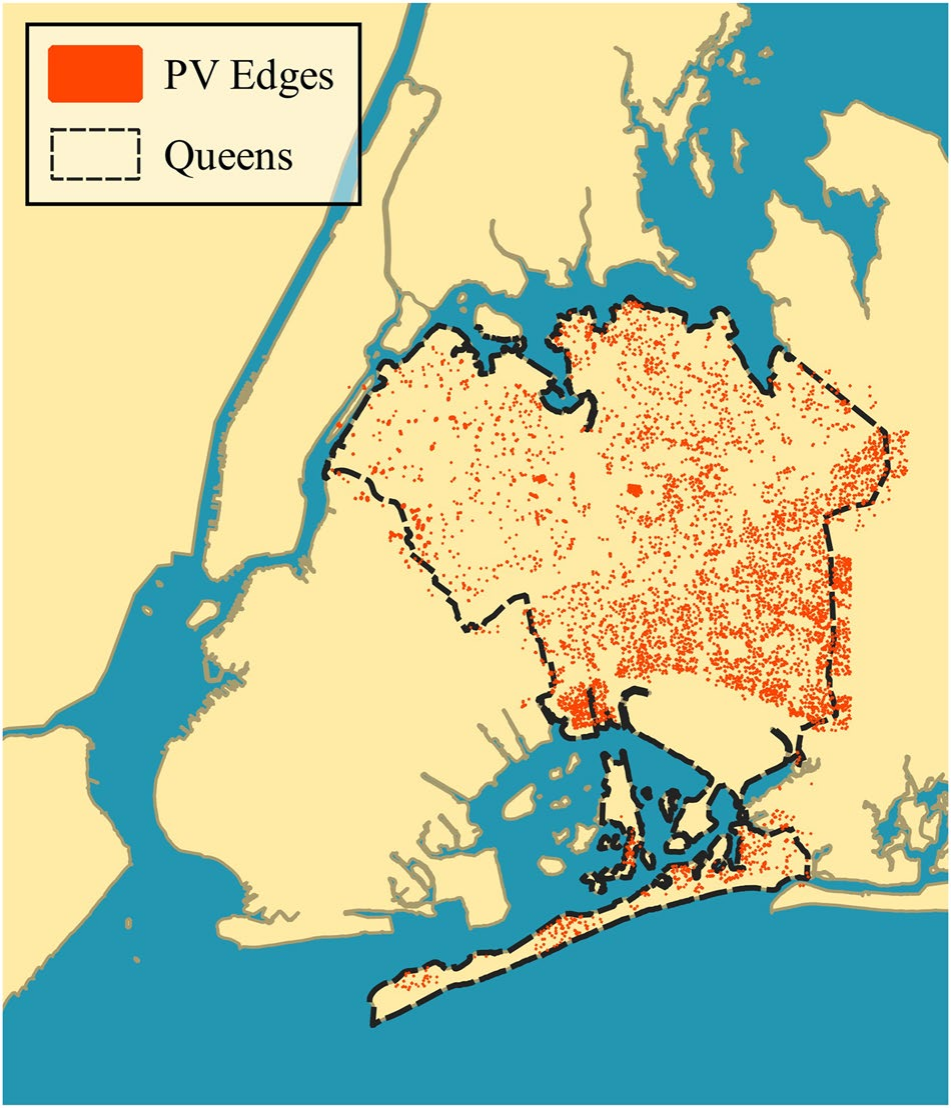
Map showing the region of interest with all PV labels.

The dataset described in this study^28^ consists of manually generated polygons outlining PV installations in Queens, New York. The dataset was constructed using publicly available orthographic projection aerial imagery (orthoimagery) from the New York State GIS Clearinghouse (https://gis.ny.gov/new-york-city-orthoimagery-downloads). The New York State GIS Clearinghouse collected aerial imagery data on a two year cycle going back to 2004 as described in their metadata (https://github.com/CityOfNewYork/nyc-geo-metadata/blob/2543257399a62fb51255c349161ad95d679a558a/Metadata/Metadata_AerialImagery.md). Data were acquired with a resolution of 6 inches (around 0.15 m) per pixel. Since 2008, imagery consists of four data channels (three color plus infrared). A map indicating the region and the distribution of labeled polygons is shown in Fig. 1. Note that some labels fall outside the municipal borough boundary, because the labeling effort was based on the footprint of the Queens-designated imagery tiles rather than the formal civil boundary of Queens.

To create the labeled polygons, the orthoimagery data were downloaded for the borough of Queens, New York, for the year 2018 from the New York State GIS Clearinghouse (https://gisdata.ny.gov/ortho/nysdop9/new_york_city/spcs/zips/boro_queens_sp18.zip). The year 2018 was chosen as it was the most recent data available when labeling began. These images comprised 667 tiles, each with a size of 5000 x 5000 pixels. The tiles were four-channel color infrared images in a JPEG2000 format. The four channel images were first converted to three channel visible images in PNG format, so that they could be opened and interpreted by users operating common computer software. However, as no changes to the image locations occured in this transition, the labels remained valid for the original imagery. These visible image files were accessed using the LabelMe computer software package (https://github.com/labelmeai/labelme). Labels were first created for each image by a team of individual annotators, who manually inspected the images one at a time and outlined visible PV instances with polygons. After all images were initially labeled, a consensus-seeking process was employed for final quality control. A team of three trained annotators formed a review committee and re-inspected each image for missed or mislabeled areas, logging any proposed changes prior to making adjustments. Confusing or ambiguous areas were discussed collectively and subjected to year-over-year imagery comparisons.

**Fig. 2:**
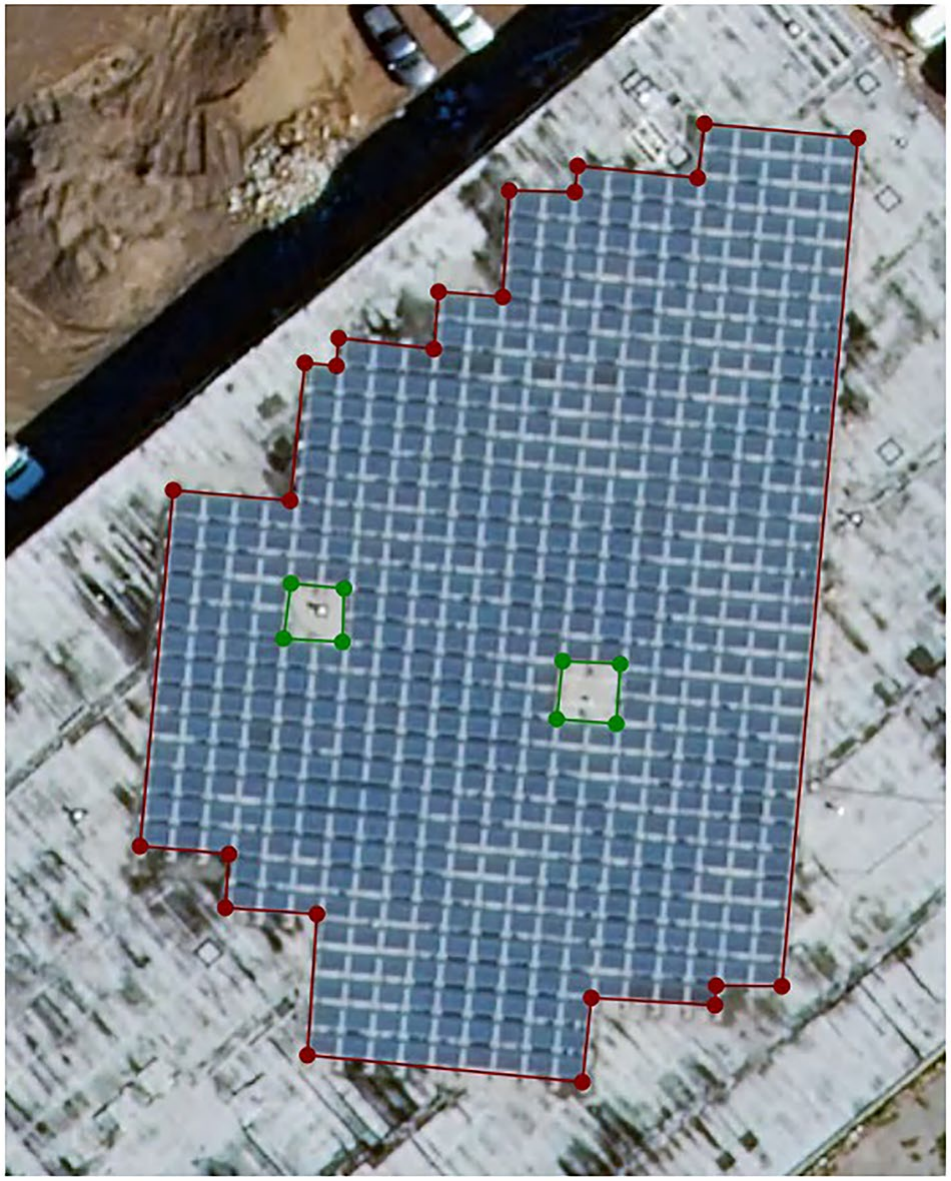
Example of pv (red) and notpv (green) polygons.

Two label classes were used: *pv* and *notpv*. The former was used to represent the outer boundary of photovoltaic arrays observed in the images. As the LabelMe package did not allow for polygons with inner rings, the use of a *notpv* class was necessary to mark the inner gaps within a larger area of PV. An example of an image showing both classes is shown in Fig. 2.

## Data Records

The dataset is available in a Zenodo repository named Segmentation Dataset of Labeled Photovoltaic Installations for Orthographic Aerial Imagery in Queens, New York^28^. The data that make up the labels are presented in three formats listed below. All annotation and label data are licensed under a Creative Commons Attribution International 4.0 license. A copy of the source imagery data used to create this dataset is provided in a zip format in the repository for reference only. For full details of the files in the source imagery dataset, please refer to the original New York Data repository https://gis.ny.gov/new-york-city-orthoimagery-downloads and its metadata (https://github.com/CityOfNewYork/nyc-geo-metadata/blob/2543257399a62fb51255c349161ad95d679a558a/Metadata/Metadata_AerialImagery.md)).

The data repository consists of two zip files. The following data organization is used by the repository:

- *NYQ18-labels.zip* - a zip file containing the the PV labels making up the new dataset. All items in this archive were created by the authors in this study.
  - *json* directory contains the polygon labels for each frame in a format readable by the LabelMe package that was used in their creation. The filenames for each JSON file correspond to the tile name from the source NYS GIS Clearinghouse dataset.
  - *mask* directory contains binary mask PNG images derived from the JSON, each with a size of 5000 x 5000 pixels, corresponding to the initial imagery size. File names correspond to the tile name from the source NYS GIS Clearinghouse dataset.
  - *shapefile* directory - contains a zipped ESRI shapefile that contains all polygons for the entire dataset. It was generated by georeferencing the JSON files back to the coordinate reference system used by the source data set. Each polygon is additionally tagged with the name of the image tile to which it corresponds. The shapefile uses the New York State Plane Coordinates, Long Island East Zone, NAD83, US foot coordinate system corresponding to EPSG:2263.
- *boro_queens_sp18.zip* a time-of-publication copy of the source imagery dataset downloaded from the New York State GIS Clearinghouse at (https://gisdata.ny.gov/ortho/nysdop9/new_york_city/spcs/zips/boro_queens_sp18.zip). This data is reproduced here for the convenience of users and reshared under the terms of CC-BY 4.0 as specified in the metadata.

## Data Overview

**Fig. 3:**
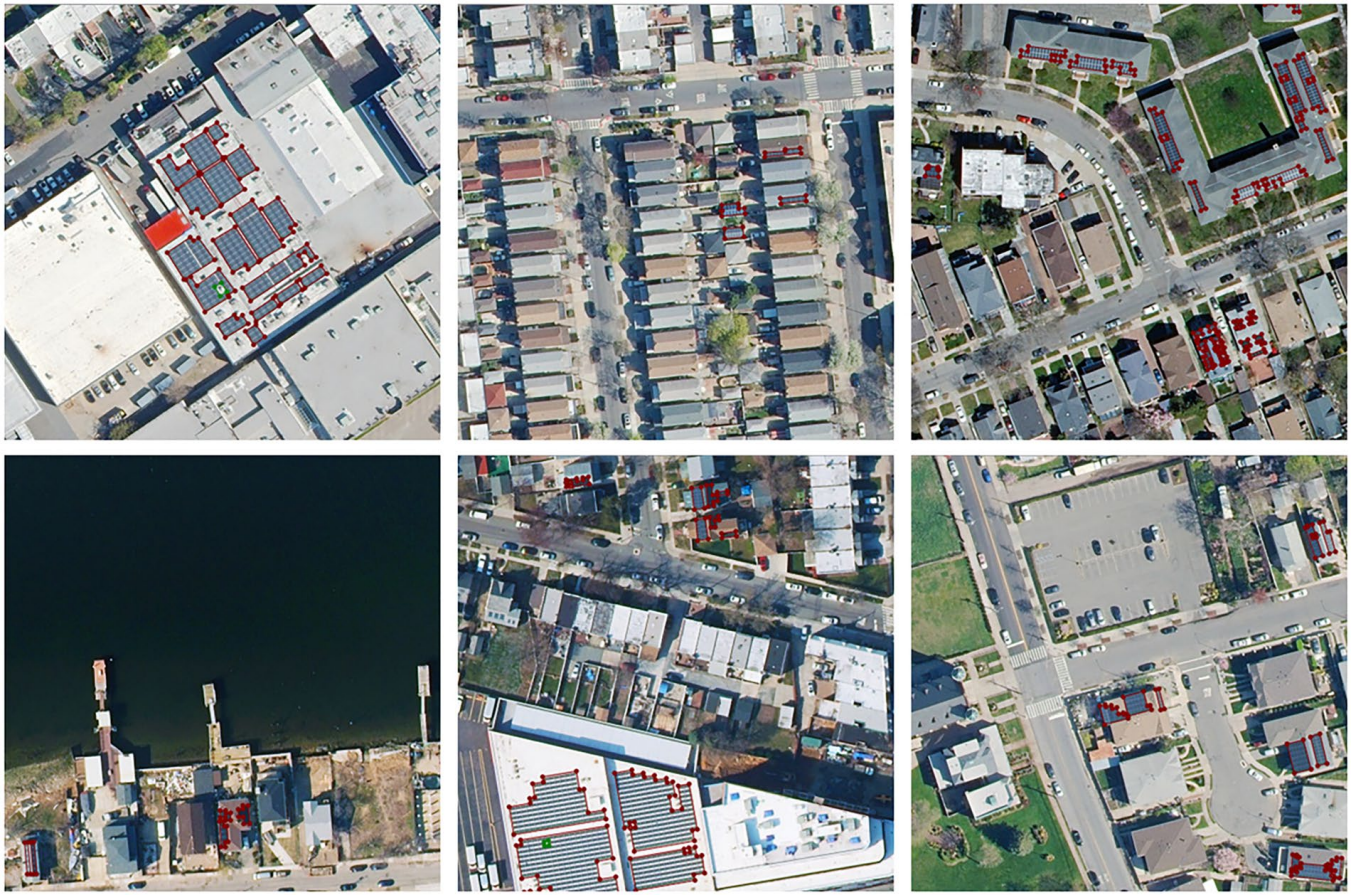
Sample images from the dataset.

Some summary statistics related to the polygon data may be found in Table 2. Note that the number of individual installations was not calculated directly, by virtue of lacking a formal definition for what constitutes a single installation. Rather, polygons representing a common installation were instead inferred by grouping nearby polygons based on a 9.1 meter spatial buffer. Some sample labeled images from the dataset are shown in Fig. 3.

**Table 2:** Summary Statistics.

Number Images	667
Image Size	5,000 × 5,000 pix
Image Resolution	6 in/pix (around 0.15 m/pix)
Image Footprint Area	282.6 km^2^
Number Polygons	14,064
Number Installations (Approx.)	5,523
Median Single Polygon Area	13 m^2^
Total Polygon Area	378,444 m^2^

## Technical Validation

As this dataset consists of human labeled images that are the result of a manual process, the primary source of uncertainty in the dataset creation falls within the realm of human error. To minimize errors and maintain consistency, each image was viewed by the labeling team four times (once on initial labeling and once more by each member of the three-member quality review team), as described in the Methods section. However, despite these best efforts, the possibility exists that the dataset contains PV arrays that were not identified (false negatives), PV arrays that were incorrectly labeled (false positives) and incorrectly drawn boundaries.

**Fig. 4:**
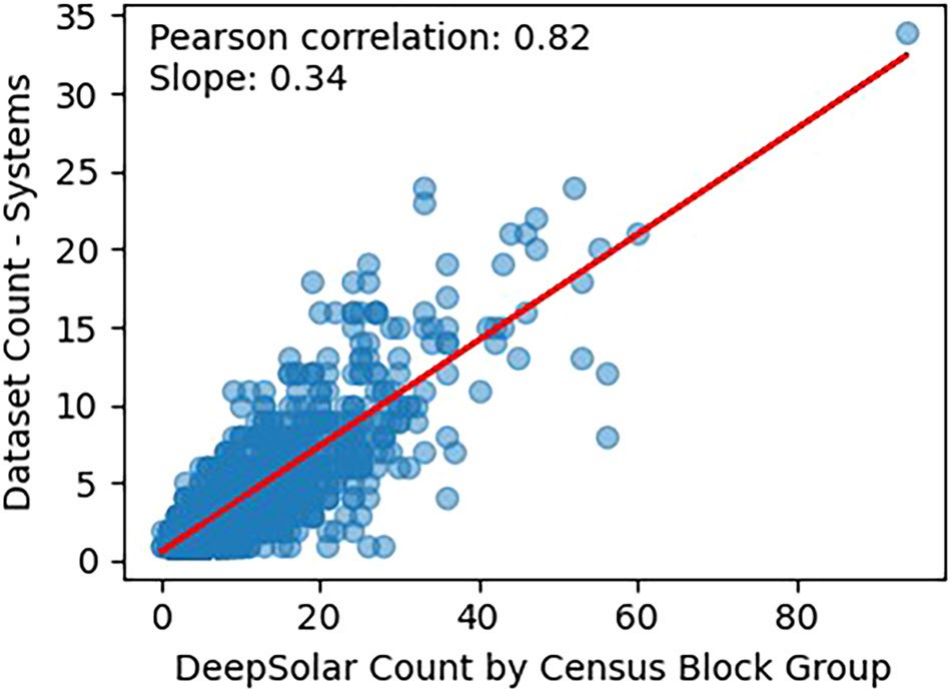
Census Block Group correlation between counted systems in the dataset (aggregated with 9.1 m buffer) and from DeepSolar-3M^9^.

We considered comparison with other datasets covering this region as a potential source of validation. Global data^7^ covering this area uses a 20 m resolution that does not provide sufficient resolution to indicate rooftop systems, and consequently was not suitable for comparison. We conducted a quantitative comparison with data from the DeepSolar-3M analysis^9^, which performed a computer vision based survey of PV at the level of counties and census block groups in the United States. We computed the number of systems within each census block in our dataset, as found by merging nearby polygons in the dataset using a 9.1 meter buffer. The county level counts do not agree directly between the two sources, which may be attributable to the difference in the year of the data. As shown in Table 2, our dataset identified 5,523 systems from 2018, compared to 13,680 identified in DeepSolar-3M in 2022. We conducted a census block group comparison of the number of systems identified by our dataset and DeepSolar-3M and found a correlation coefficient of around 0.8, as shown in Fig. 4. We note that while DeepSolar-3M does not represent a source of ground truth due to being machine labeled, this comparison shows a good degree of agreement between the data, demonstrating that these two datasets identify PV in similar locations.

**Fig. 5:**
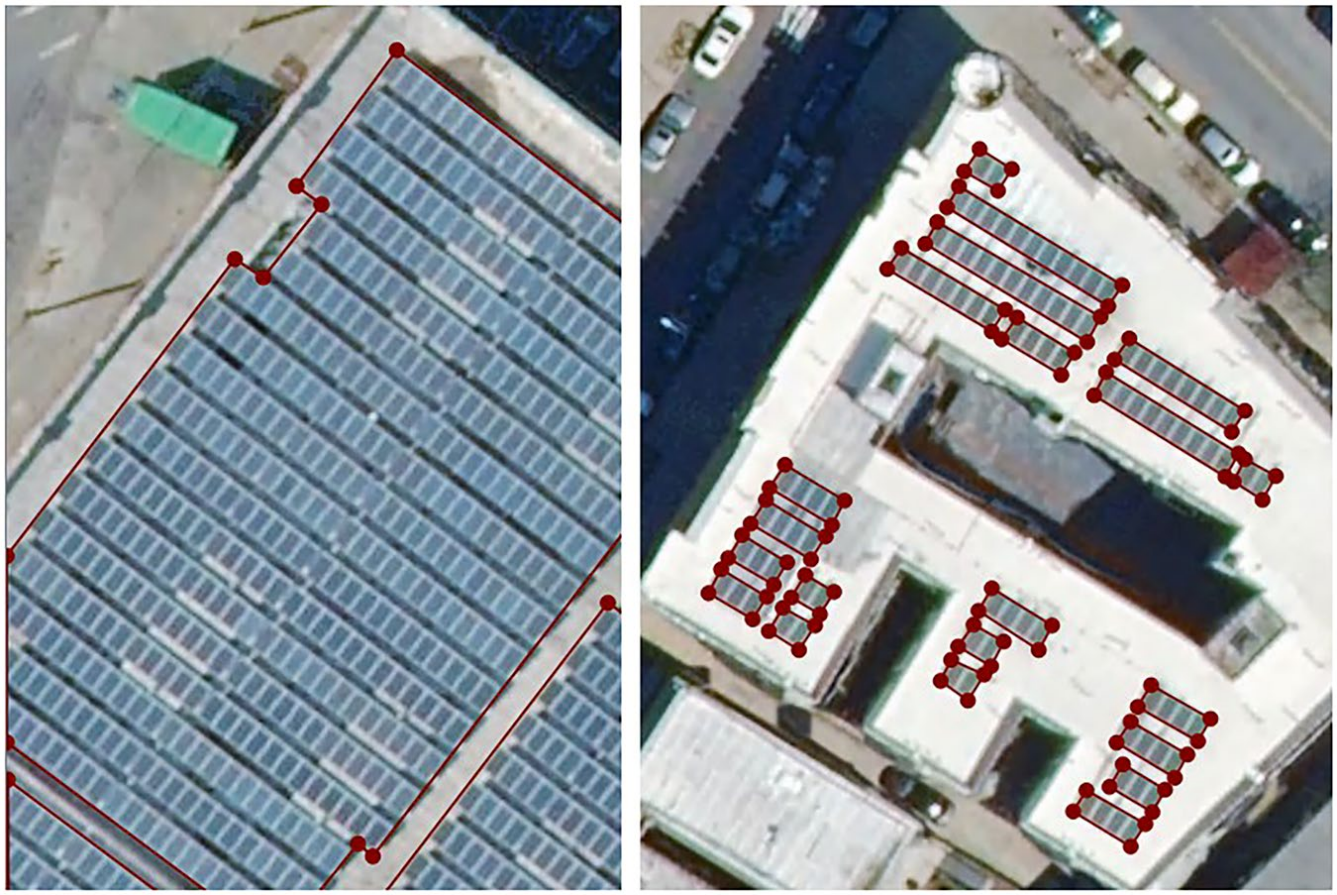
Contrasting examples of rows being identified in whole vs. being identified individually due to wider spacing.

Some known edge cases that were encountered during dataset creation for which labeling proved difficult or uncertain for human labelers are described here. PV array and module boundaries were drawn as faithfully to the outer edge of the PV as possible, but wide variation in the amount of spacing between rows and modules were observed throughout the data. Where it was reasonable to do so, we opted to select panels in individual rows to produce polygons that selected only actual PV modules, but there remains some ambiguity and labeler judgment in the distinction, because it was not possible to objectively quantify row spacing using the tools available. Example cases where module rows were and were not separated are shown in Fig. 5.

Some images contained cases that were difficult to categorize, or may remain uncertain or ambiguous, even to a trained human labeler. Examples included the presence of tar paper rooftops, highly reflective panels, greenhouses or similar patio roofs, awnings and modules with visually indistinct frames. On the other hand, parking spaces and crosswalks were typically distinguishable by human labelers though these sometimes present challenges for machine trained systems. Communication between the labelers took place throughout the entire labeling process to share experience and discuss ambiguous cases, with special attention paid to reaching agreement during the quality review process. To quantify the converging interpretation, a log was maintained of editing done on each of the quality review passes, indicating any edits that were proposed. Referring back to the 667 original images, a total of 353 images were edited in some way during the quality review process. At the conclusion of the process, only 10 images had received notes from all three reviewers, indicating that the final dataset reflects a state of strong consensus that had been reached by the process.

**Fig. 6:**
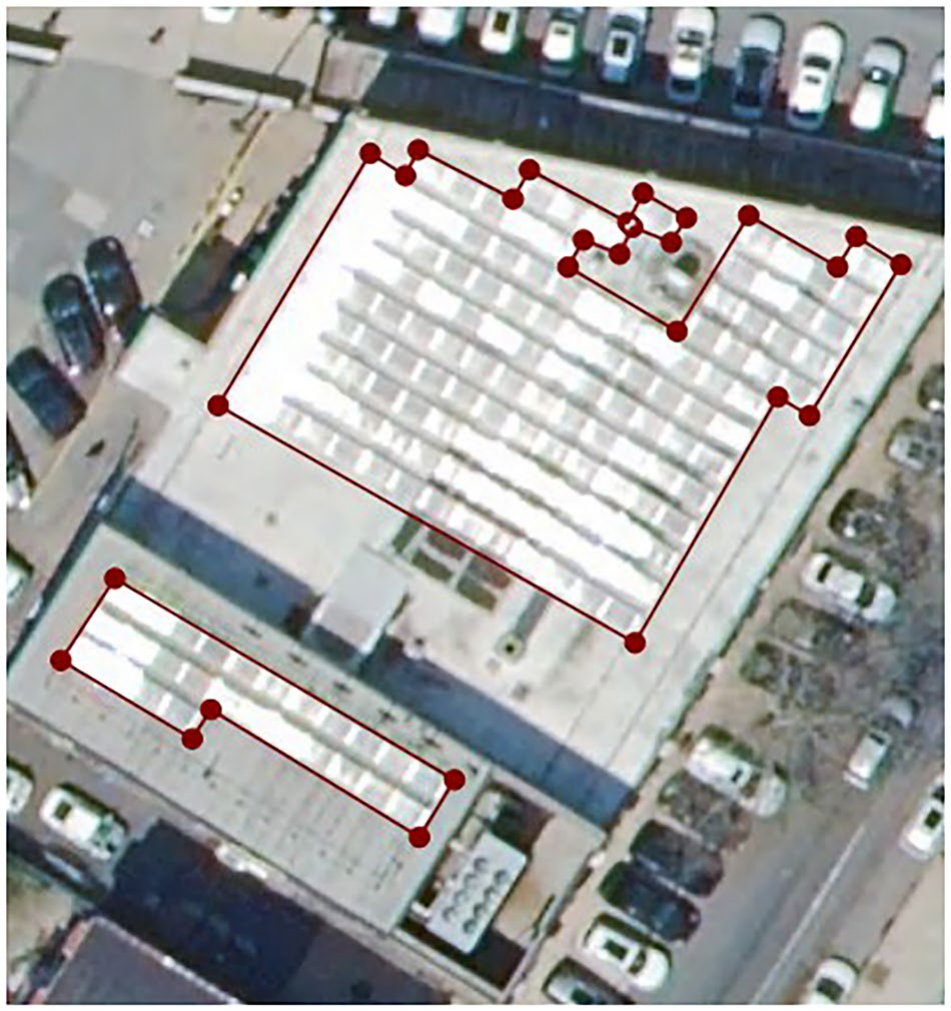
Example of bright specular reflection from modules.

**Fig. 7:**
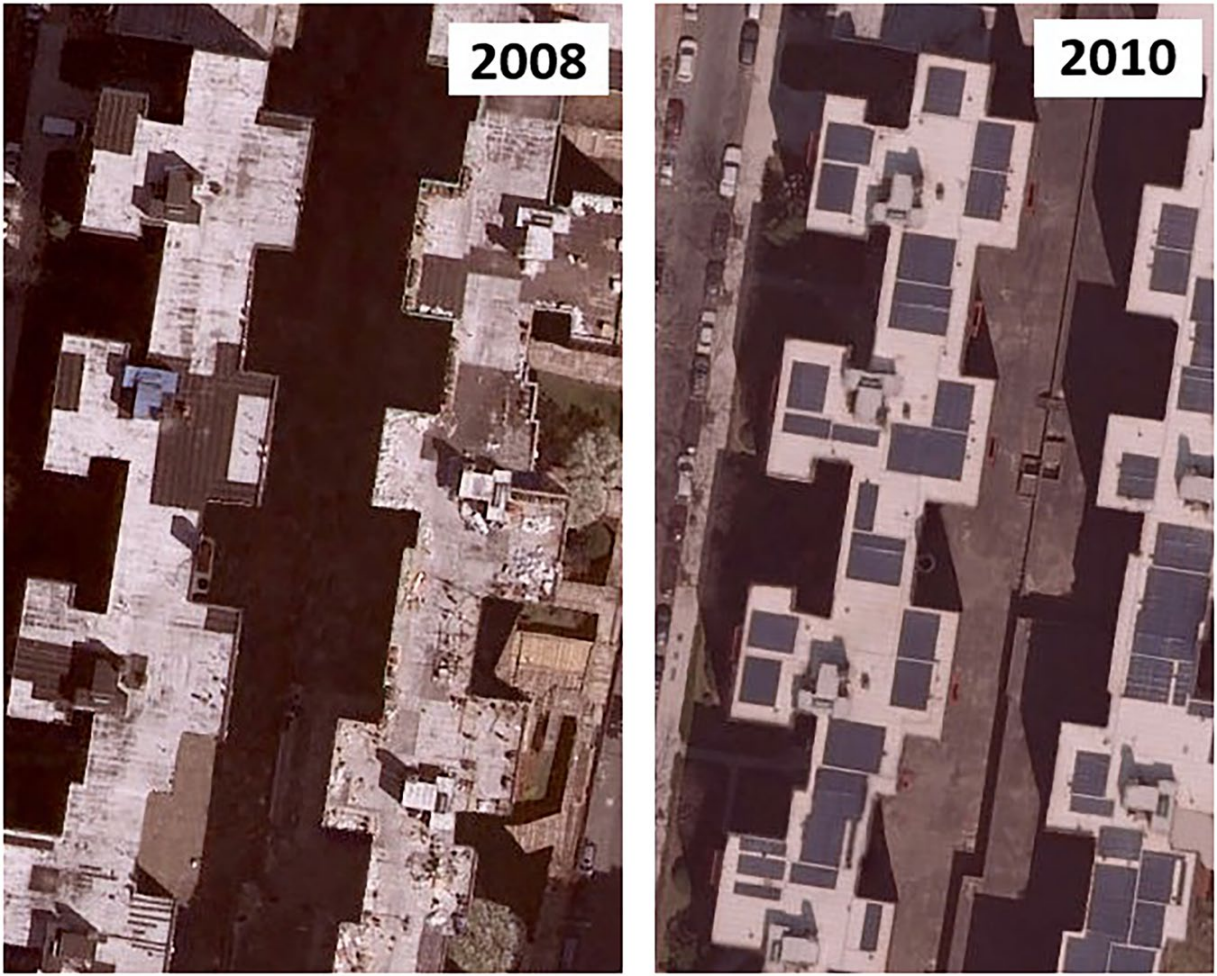
Example of changes from year to year that are at the limit of labeler capability to distinguish with certainty. Left and right show the same location for different years in the archive.

Due to the availability of multiple years of data in the source data archive, when ambiguity in the presence of PV panels existed, images for alternate years were consulted to improve judgment where possible. Consulting alternate years was particularly useful for discerning cases where solar reflections from the modules were observed for the 2018 data, such as in Fig. 6, where alternate years typically did not experience an identical specular reflection. Despite this, some ambiguous cases remained even when considering multiple years of data. For example, the rooftops shown in Fig. 7 have dark rectilinear shapes that appear en masse between 2008 and 2010 images and remain essentially unchanged in all images since. On the other hand, they lack visible module edges that would unambiguously identify them as solar arrays and represent a judgment call on the part of the labelers. In the case of the example shown here, the regions are labeled as PV in the dataset. The number of these completely indeterminate cases encountered in the final quality review were few, but we do note that due to the absence of an available cross-check of rooftop solar installation data for this location, this practical limit to human discernment of the imagery is a limitation of manually labeled data validation.

## Data Availability

The dataset is available at the repository DOI (10.5281/zenodo.15084216)^28^.

## Code availability

Auxiliary python code for the project is available in a GitHub repository named *pvlabels_queens* (https://github.com/jranalli/pvlabels_queens). The provided codes can be used to convert the LabelMe JSON files into binary mask images, to aggregate the polygons from the JSON files into the overall shape file, and to convert the dataset’s raw JPEG2000 images into PNG files for more convenient use with many common software tools. Since the native tile size of 5000 × 5000 pixels is quite large for most machine learning approaches, users may wish to consider the *split-image* python package (https://pypi.org/project/split-image/) that can be used to automate generating tiles from large images as described in the *pvlabels_queens* repository’s README.
